# A Novel Deep Learning-Based Approach for Segmentation of Different Type Caries Lesions on Panoramic Radiographs

**DOI:** 10.3390/diagnostics13020202

**Published:** 2023-01-05

**Authors:** Burak Dayı, Hüseyin Üzen, İpek Balıkçı Çiçek, Şuayip Burak Duman

**Affiliations:** 1 Department of Restorative Dentistry, Faculty of Dentistry, Inonu University, Malatya 44280, Turkey; 2 Department of Computer Engineering, Bingol University, Bingol 12000, Turkey; 3 Department of Biostatistics and Medical Informatics, Faculty of Medicine, Inonu University, Malatya 44280, Turkey; 4 Department of Oral and Maxillofacial Radiology, Faculty of Dentistry, Inonu University, Malatya 44280, Turkey

**Keywords:** caries diagnosis, convolutional neural network, dental panoramic radiographs, deep learning

## Abstract

The study aims to evaluate the diagnostic performance of an artificial intelligence system based on deep learning for the segmentation of occlusal, proximal and cervical caries lesions on panoramic radiographs. The study included 504 anonymous panoramic radiographs obtained from the radiology archive of Inonu University Faculty of Dentistry’s Department of Oral and Maxillofacial Radiology from January 2018 to January 2020. This study proposes Dental Caries Detection Network (DCDNet) architecture for dental caries segmentation. The main difference between DCDNet and other segmentation architecture is that the last part of DCDNet contains a Multi-Predicted Output (MPO) structure. In MPO, the final feature map split into three different paths for detecting occlusal, proximal and cervical caries. Extensive experimental analyses were executed to analyze the DCDNet network architecture performance. In these comparison results, while the proposed model achieved an average F1-score of 62.79%, the highest average F1-score of 15.69% was achieved with the state-of-the-art segmentation models. These results show that the proposed artificial intelligence-based model can be one of the indispensable auxiliary tools of dentists in the diagnosis and treatment planning of carious lesions by enabling their detection in different locations with high success.

## 1. Introduction

Dental caries is one of the most common chronic diseases and affects more than three billion people worldwide [1]. Clinicians need to detect caries at the enamel and dentin levels before these turn into irreversible lesions [2]. Dental radiographs are an essential diagnostic tool for helping clinicians to diagnose caries. One type of extra-oral radiograph is the digital panoramic, which is widely used in many areas of dentistry and provides safer, more accurate and relatively cheaper results today [3]. Contrary to intra-oral radiographs, it has minimal spatial resolution and is open to significant, unpredictable geometric distortion [4]. For this and many other reasons (different tooth anatomical morphologies and restorative forms, etc.), even though dental radiography and explorer (also known as a dental probe) are often employed and considered very reliable diagnostic techniques for the identification of dental caries, a large proportion of screening and final diagnosis frequently relies on empirical evidence [5]. However, this approach is highly subjective and in crowded clinics mistakes or underdiagnoses may happen depending on the knowledge and focus of the clinicians. This can be avoided by integrating artificial intelligence software with radiographs to assist the clinician during the diagnosis and treatment phases.

Artificial intelligence (AI) is a generic phrase used to describe the development of computer systems that can carry out functions that typically require human intellect. The applications of AI in dentistry are primarily virtual, using AI algorithms to separate lesions from healthy structures, rank risk variables and simulate and assess future outcomes [6]. Convolutional neural networks (CNNs) have been effectively used in recent years to clarify numerous healthcare difficulties involving various forms of medical imaging. Skin cancer classification from dermoscopic pictures, breast cancer detection from thermal images, Alzheimer’s disease diagnosis using SPECT data and automated identification and quantification of COVID-19 from chest computed tomography records are a few new techniques employing CNNs [7,8,9,10].

Radiology is a key part of the diagnostic process in dentistry. Every year, a considerable number of images, including panoramic, bitewing, periapical and cephalometric radiographs, are acquired in dental radiology [11]. Given this massive number of picture records, CNNs appear to have enormous clinical evaluation and diagnostic potential. Deep learning researchers have just begun to investigate this potential in the realm of dental radiography. CNNs have been utilized effectively in periapical radiographs to diagnose periodontal bone loss [12], bitewing radiographs to detect carious lesions [13] and panoramic radiographs to detect apical lesions [14]. A thorough examination of the use of CNNs in dental radiology is provided. Furthermore, it is feasible to envision how the application of artificial intelligence in dentistry might yield gratifying results, particularly in the field of caries diagnostics [15].

Briefly, in connection with various medical and dental practices, the deep learning system, one of the most promising artificial intelligence models, has been developed [16,17]. Deep learning systems can automatically classify datasets and, with the aid of multilayer CNNs, they can learn in-depth about the features present in the data [18].

Tooth segmentation is crucial for the automated detection of tooth-related disorders on dental radiologic images, but manual annotation is a laborious and time-consuming operation. Therefore, the first (and most difficult) step in creating automated interpretable diagnostic procedures for dental images is the automation of tooth segmentation. CNN has recently been widely used in dentistry to overcome the constraints associated with traditional segmentation algorithms [19,20,21].

The application of CNNs in dentistry, particularly for the detection of caries, is a novel technique [13]. The aim of this study is to develop a new deep-learning system for the segmentation of different types of carious lesions on panoramic radiographs. In this direction, the Dental Caries Detection Network (DCDNet) architecture was developed to detect different types of caries lesions and performance was compared with other existing models.

## 2. Material and Methods

### 2.1. Image Dataset

Inonu University Non-Interventional Clinical Research Ethics Board (number 2022/3774) authorized the study protocol. The principles of the Helsinki Declaration were followed. The study included anonymous panoramic radiographs obtained from the radiology archive of Inonu University Faculty of Dentistry’s Department of Oral and Maxillofacial Radiology from January 2018 to January 2020. The age range of the patients to whom the radiographs belonged was between 14 and 80. The percentage of males and females was 29.5 and 70.5, respectively. Radiographs were obtained using the Planmeca Promax 2D Panoramic System (Planmeca, Helsinki, Finland) with image acquisition parameters of 68 kVp, 14 mA and 12 s.

### 2.2. Image Evaluation and Labeling

**Figure 1:**
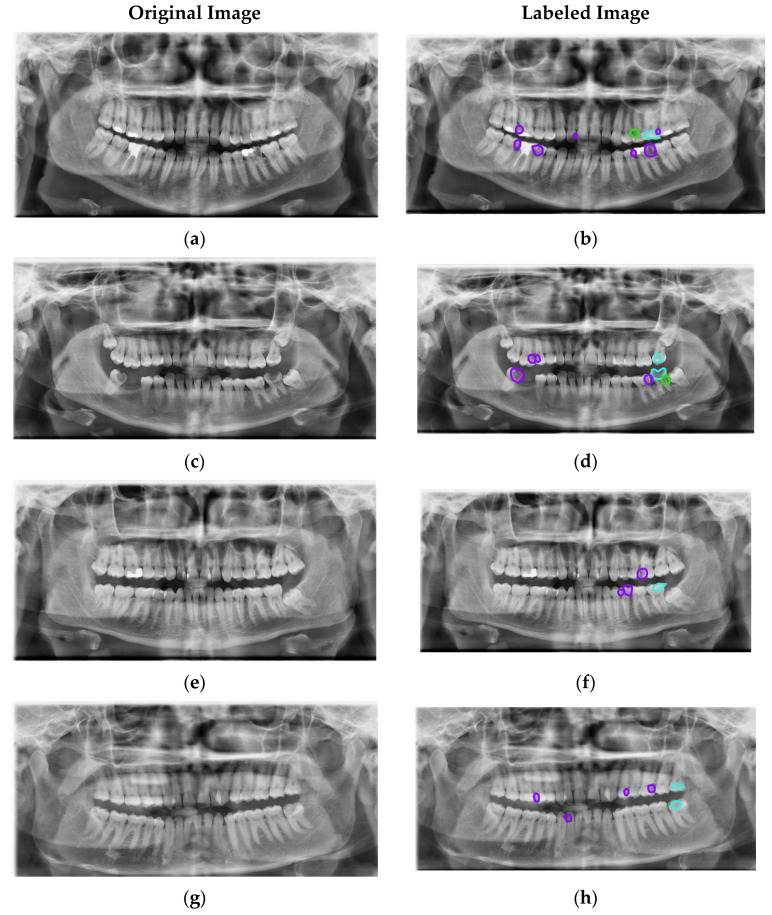
Example images (**a**,**c**,**e**,**g**) and labels (**b**,**d**,**f**,**h**). 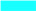 Type I (Occlusal caries). 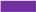 Type II (Proximal caries). 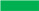 Type III (Cervical caries).

Radiographs with artifacts and in which no caries could be detected were not included in the analysis. Occlusal caries detected on the panoramic radiographs were labeled as Type I, proximal caries as Type II and caries in the cervical region as Type III with the Plainsight Software System (San Francisco, CA, USA). Labeling was performed with the joint decision of an 8-year restorative dental specialist and a 5-year oral and maxillofacial radiologist. In case of instability, the relevant images were excluded from the study. As a result, 504 panoramic radiography images were used in experimental studies and 746 occlusal caries (Type I), 1627 proximal caries (Type II) and 378 cervical caries (Type III) labels were made in these images (Figure 1).

### 2.3. Construction of the Models

Pre-trained network architectures are used to ensure the high performance of our proposed model. In this study, MobileNetV2, VGG16, ResNet50 and EfficientNet and Inception network architectures, which have recently provided high performance, have been used. In addition, the decoder module of the proposed model was inspired by the Feature Pyramid Network. The architectures used are briefly described below.

#### 2.3.1. MobileNetV2

MobileNetV1 is a convolutional architecture designed for low-cost or mobile devices that minimizes network cost and size. This has resulted in the ease of use of image processing and deep network categorization in mobile devices. The MobileNetV2 model was based on the MobileNetV1 and tackled difficulties relating to nonlinearities in the model’s thin layers containing building blocks [22]. The MobileNetV2 model can perform classification, segmentation and object identification and adds two additional features to its predecessor. The first is that some bottlenecks might appear linearly between layers; the second is the development of shortcuts between bottlenecks [23].

The MobileNetV2 design includes depth wise (dw) separable filters and combination stages. This model employs a deep convolution filter for each layer input with a resolution of 1 × 1 pixel. Depth wise separable convolutional filters investigate inputs by dividing them into two distinct layers. This reduces both the model’s speed and cost. The features obtained by filter separation are combined in the combining stages and a new layer is formed. Batchnorm and Rectified Linear Unit (ReLU) linearity are used in constructing the MobilNetV2 model [24].

#### 2.3.2. VGG16

In 2014, Zisserman and Simonyan presented VGG16 as a VGGNET network structure. It is a more extensive network constructed on top of the AlexNet network. It can more correctly describe the data collection properties while recognizing and classifying images and outperforms other methods when dealing with big datasets and complicated backdrop recognition tasks. The network topology consists of three fully connected layers, 13 convolutional layers and five pool layers. Compared to other networks, the VGG16’s 13 convolutional layers employ a medium-sized 3 × 3 matrix with a moving step of 1. The number of convolution kernels steadily rose from 64 in the first layer to 128 to 256 in the second layer and then to 512 in the final layer. The convolution kernel in the pooling layer has a size of 2 × 2 and a step size of 2. With a convolution kernel size of 5 × 5, it outperforms other networks in terms of extracted features [25].

#### 2.3.3. ResNet50

The name ResNet is an abbreviation for residual neural networks. It is an improved version of CNN with a large number of convolutional neural networks. ResNet tries to solve saturation and accuracy loss in the deep CNN training process [26]. ResNet50 is a residual network with 50 layers. Having transitions between layers deepens the network in ResNet models. The deterioration that may occur in the deepening network is prevented thanks to these transitions. Moreover, ResNet models employ a mixture of multiple-sized convolution filters to counteract degradation and minimize training time due to deep structures. These models use blocks called bottlenecks for rapid training [27].

#### 2.3.4. EfficientNet

EfficientNet is an algorithm that uses convolutional neural networks. EfficientNet has an architecture that focuses on improving the efficiency of models as well as their accuracy. EfficientNet consists of 8 different models between B0-B7. The model scales use three separate parameters. These parameters are depth, width and resolution. The depth parameter measures how deep the networks are, while the width parameter is the number of neurons in the layers. The resolution parameter expresses the resolution of the dataset on which the model will be trained. Within the scope of the study, the classification process was carried out with EfficientNet, unlike existing CNN models, which employ a novel activation function termed Swish rather than a ReLU activation function [28]. EfficientNet also provides more efficient outcomes in other cutting-edge models by consistently scaling depth, breadth and resolution while scaling down the model. The first stage in the compound scaling approach is to find a grid that will assist in determining the relationship between the various scaling dimensions of the baseline network while working with a fixed resource limitation. Thus, an appropriate scaling factor for the depth, breadth and resolution parameters is calculated. The coefficients are then applied to scale the baseline network to the desired target network [29].

#### 2.3.5. Feature Pyramid Network

A Feature Pyramid Network (FPNet) is a fully convolutional feature extractor that takes as input a single-scale image of any size and outputs proportionally sized feature maps at multiple levels. This process does not depend on the architecture of the convolutional backbone, so may be used as a universal approach for building feature pyramids within deep convolutional networks for applications such as object recognition [30].

A bottom-up and a top-down path are used to build the pyramid. The bottom-up pathway is the backbone of ConvNet’s feedforward computation. It builds a feature hierarchy out of feature maps at different scales with a scaling step of 2. For each step of the feature pyramid, one pyramid level is defined. As a reference set of feature maps, the output of the last layer of each stage is used [31]. The top-down path gives the impression of higher resolution features by upsampling feature maps from higher pyramid levels that are geographically coarser but have better meaning. Then, using lateral connections, these traits are added to traits from the bottom-up pathway. Each lateral link is made up of equal-sized feature maps from the top-down and bottom-up pathways. The bottom-up feature map has lower-level meanings, but its activations have been more accurately localized because there was less subsampling [32].

### 2.4. Proposed Network Architecture

**Figure 2:**
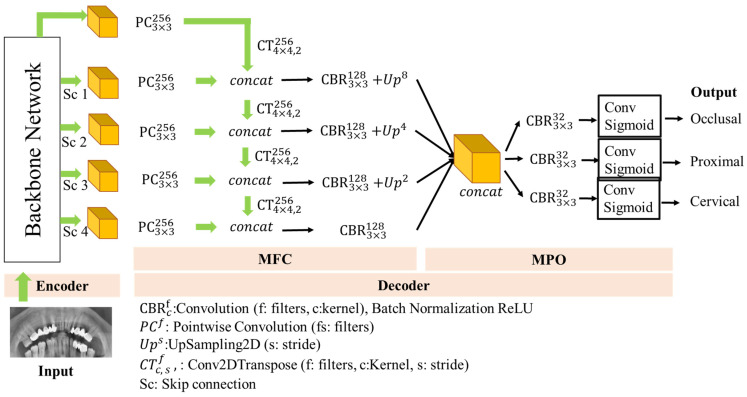
Proposed deep architecture.

In this study, Dental Caries Detection Network (DCDNet) architecture is proposed for dental caries detection. This network architecture is encoder-decoder based as shown in Figure 2. The Encoder part consists of pre-trained backbone network architectures such as VGG16, MobileNet and EfficientNet. In the proposed model, initial features are obtained from these backbone networks. The decoder part of the proposed DCDNet network consists of two parts, the Multi-level Features Concatenation (MFC) module and the Multi-Predicted Output (MPO) Block. It is fed with features from different levels of the backbone network in the MFC model. In this section, new powerful feature maps are obtained for caries detection. The second part of the decoder generates the prediction map for the three caries types from the final feature map in the MPO block. For this process, the final feature map proceeds in three ways and convolution and sigmoid activation are applied.

#### 2.4.1. Encoder

Pre-trained backbone networks are used in the encoder part of the proposed model. The backbone networks used in experimental studies are EfficientNet, ResNet50, VGG16, Inception-V3 and MobileNetV2 networks, respectively. Each backbone network has connection points, as shown in Table 1. Features taken from these ports are transferred to the decoder section.

The primary purpose of using backbone networks shown in Table 1 is to obtain initial features for a limited dataset. In this way, a more efficient training procedure takes place.

#### 2.4.2. Decoder

The decoder of the proposed model consists of two stages; the first is the feature integration module, MFC. The MFC model was inspired by the FPNet model. As shown in Figure 2, the MFC module consists of four levels. ConvBNReLU blocks have been applied to the input features at the beginning of each level. Then the resulting output was combined with the skip connection. In the merging process, the Pointwise Convolution (PC) layer was primarily applied to the feature maps. In this way, the two feature maps were brought to the same size. The element-based sum operation was then applied to these feature maps. These processes were repeated for the four levels of the MFC.

At the last level of MFC, feature maps of all levels were combined. In this way, essential details obtained in the intermediate layers were preserved. The combined feature maps were transferred to the MPO unit to obtain dental caries prediction output.

The MPO block divided the final feature map into three paths, as shown in Figure 2. Each path represents one type of caries. Here, the final feature map, like Unet and FPN, were not used directly. The main reason for this is to prevent the interaction of caries types that are very closely related to each other. Thus, it detects with higher scores than other single-output architectures.

### 2.5. Training Procedure

In transfer learning architectures based on the CNN model, high-performance results are achieved through learning transfer. These models have provided significant improvements, especially in the field of image processing. The EfficientNet architecture and other deep learning models were trained using the transfer learning approach.

The initial parameters of the backbone networks used in the proposed DCDNet network were used with ImageNet-trained parameters. In addition, the other layers in the decoder section were initialized with random values. Detailed layer structures, connections and other details of the proposed DCDNet architecture are given in Table 2.

The Binary Cross-Entropy loss function was used in the training of the DCDNet network, defined as in Equation (1).

(1) $$L_{k}=-\overset{M}{\underset{i,j}{\sum }}y_{i\times j}log\left(P_{i\times j}\right)+\left(1-y_{i\times j}\right)log\left(1-P_{i\times j}\right)\;L=\frac{1}{N}\overset{N}{\underset{k}{\sum }}L_{k}$$

Here, $y_{i\times j}$ and $P_{i\times j}$ represent the actual and predicted values of the pixel at position ***i*** *× **j***, respectively. ***M*** and ***N*** represent the total number of samples and the number of caries types, respectively. The $L_{k}$ value shows the mean error value obtained for k-type (k∈{1, 2, 3}) caries and the ***L*** result error value.

## 3. Results

In this section, experimental studies have been carried out to analyze the caries detection performance of the proposed DCDNet network. Firstly, the backbone networks of the proposed model were compared. Then, the proposed model was compared with other models, such as Unet and FPNet.

### 3.1. Application Details

**Figure 3:**
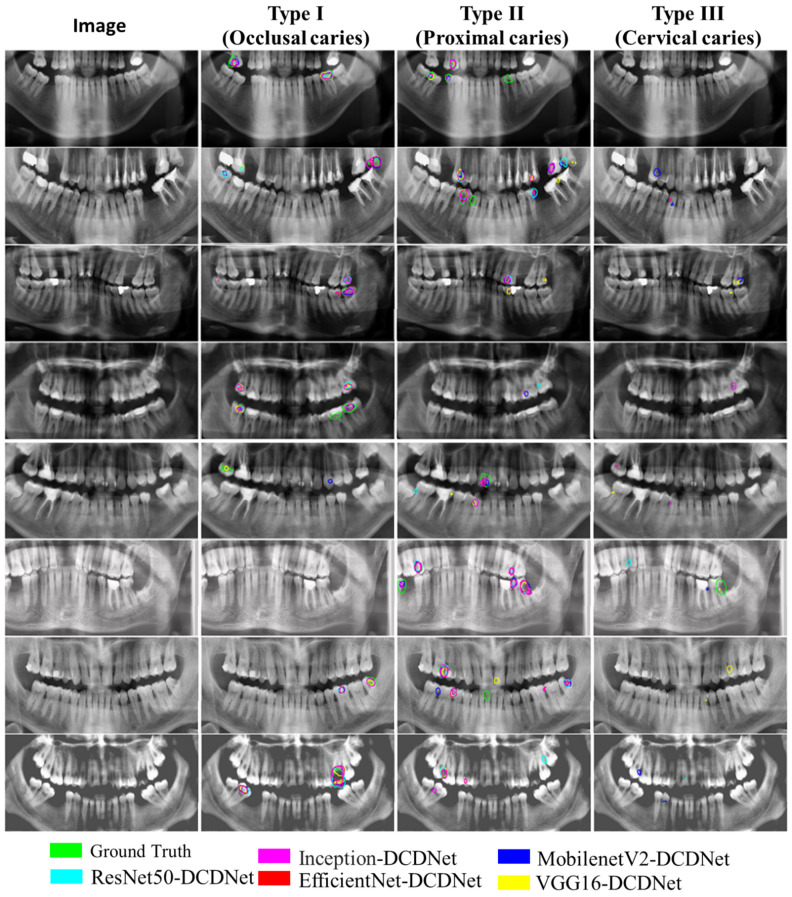
Sample images from the dataset and the prediction outputs of the models.

To evaluate the DCDNet network, the Dental Caries dataset produced in this study was used. Panoramic radiography images in the dataset are 900 × 1700 in size. As seen in Figure 1 in these high-resolution images, there are external parts, such as the other parts of the lower and upper jaw apart from the teeth. These external parts, except the teeth, are unimportant for caries detection. Therefore, the images were cropped at 540 × 1300 size with reference to the middle part of each image covering the teeth (Figure 3). Then, these cropped images were reduced to 256 × 512 size to give the input of the network architecture.

For the training phase, 75% of the panoramic radiography images that marked dental caries and their types were used. The remaining images were used for testing. The training and testing processes of the proposed model were carried out using the TensorFlow-Keras library in a python environment. In training the model, the Batch size was set to 8, the learning rate to 0.001 and the training epoch to 100. Adam’s optimization method was employed to update the network parameters. All experiments were performed under the Ubuntu 18.04 system using the Intel Xeon CPU, 128GB RAM and Nvidia P40 (24 GB).

In experimental studies, Precision, Recall, F1-score and mIoU (mean intersection over union), and metrics were used to evaluate the proposed model performance. This metric is defined as follows:

(2) $$\mathrm{Precision}=\frac{TP}{TP+FP}\;\mathrm{Recall}=\frac{TP}{TP+FN}\;F1-\mathrm{score}=\frac{2\times \mathrm{precision}\times \mathrm{recall}}{\mathrm{precision}+\mathrm{recall}}\;\mathrm{mIoU}\;=\frac{pr\cap^{\;}GT}{pr\;\cup^{\;}GT}$$

where *TP*, *FP* and *FN* refer to true positives, false positives and false negatives, respectively. *GT*, *pr*, mIoU and F1-score denote the ground truth, the prediction map, mean intersection-over-union and F1-score metric, respectively.

### 3.2. Dental Caries Detection Results

The proposed DCDNet model consists of a pre-trained backbone network, MCF and MPO structures. Four different models were chosen as the backbone network. In this way, the highest performances are aimed for. The results obtained with the different backbone networks of the DCDNet network are given in Table 3. In addition, sample images from the dataset and the estimation results of the models are given in Figure 3.

As can be seen in Table 3, the highest performance was obtained with the ResNet50-DCDNet architecture for the average F1-score values. In addition, an average of F1-score (62.67%) was achieved when the EfficientNet backbone network, which has achieved successful performance in many areas and recent articles, was used. On the other hand, on average, the lowest F1-score was obtained with the VGG16-DCDNet network.

Detailed performance results of the proposed models are given in Table 4.

When considering each type of caries separately in Table 4, the methods yielded a precision, recall and F1-score of over 60% for Type I and Type II caries, while Type III gave generally low results.

In the image results given in Figure 3, it was observed that the proposed models produced effective results for Type I and Type II caries detection. However, the proposed DCDNet model for detecting Type III caries remained weak.

### 3.3. Comparison of the Proposed Model with Other Methods

This section compares the proposed DCDNet network with other Unet, FPNet, Mobile-UNet and Eff-Unet models in the literature. For comparisons, the same experimental studies were performed using Unet, FPNet, Mobile-UNet and Eff-Unet networks. The results obtained in the experimental studies are given in Table 5.

As can be seen in the results given in Table 5, Unet, FPNet, Mobile-UNet and Eff-Unet models produced results below 20%.

### 3.4. Comparison of Models in Terms of Time Consumption

The DCDNet network proposed in this study produced effective results for caries detection. As shown in the previous sections, it produced high performance against other models thanks to its three-output structure. In this experimental study, the comparison of the proposed model in terms of time consumption is structured. The times for each model to process an image are given in Table 6 in seconds and milliseconds.

As seen in Table 6, the time consumption of DCDNet models varies between 80.0 and 98.3 milliseconds. The fact that the proposed DCDNet models have three outputs requires more time compared to other models. On the other hand, single-output models Mobile-Unet, Unet, Eff-Unet and FPNet have become more economical. However, compared to the results given in Section 3.3, the success of these models is quite low. In comparison, the DCDNet models, although costlier in terms of time consumption, achieved much higher success (see Section 2 and Section 3) compared to other models.

In the time comparison of DCDNet models, the fastest model was the Mobilenet-DCDNet model. In addition, this model provided the highest scores for Type 1 caries detection, as seen in the results in Table 3. However, this model produced lower results than the ResNet50-DCDNet model in detecting other types of caries. On the other hand, the ResNet50-DCDNet model required 0.093 s to process an image. Although the ResNet50-DCDNet mode is costly in terms of time consumption, it is the model with the highest average F1 score (Table 3). As a result, less than 0.1 s is required to process an image on all recommended DCDNet models. These results are sufficient for the detection of dental caries.

## 4. Discussion

Accurate, fast and timely diagnosis of dental caries is vital for both the physician and the patient in terms of restoring teeth without further treatment. Advanced treatments, besides causing time and economic loss, may adversely affect the success and the duration of the tooth in the mouth. The availability of automatic detection software that will help and guide physicians in the detection of dental caries and other pathological conditions will add a different dimension to dentistry. In the present study, a deep learning model used to segment dental caries on panoramic radiographs successfully detected occlusal and proximal caries.

To evaluate the performance of artificial intelligence programs, studies on root morphology [18,33], automatic tooth detection and numbering caries detection, [34,35,36] and classification on panoramic radiographs [37,38] were carried out. Caries detection and classification studies with artificial intelligence in panoramic radiographs are limited in number. Lee et al. [39] detected dental anomalies, including the category of dental caries, using artificial intelligence on panoramic radiographs. The study divided dental caries into four groups according to their clinical features: dental caries, cervical caries, proximal caries and secondary caries. In this study, we examined caries in three groups: occlusal caries (Type I), proximal caries (Type II) and cervical caries (Type III).

In this study, MobileNetV2, VGG16, ResNet50 and EfficientNet and Inception network architectures, backbone networks of the DCDNet network architecture which is known to have highly successful performance in recent studies in the segmentation and classification of dental caries, were compared. As a result of the comparison, the ResNet50-DCDNet network architecture gave the highest F1-score (62.79%). Then, the ResNet50-DCDNet network architecture was compared with the Unet, FPNet, Mobile-UNet and Eff-Unet models. As a result, the compared models showed a very low F1-score (highest Eff-Unet: 15.69%) compared to the model we proposed. The main reason for this may be that these models, which are the only way of distinguishing between softmax and caries, cannot provide adequate detection by not being able to distinguish varieties of caries, since the types of caries in the study are similar to each other. The DCDNet network, whose development is based on this problem, produces separate outputs with an MPO block for each caries as shown in Figure 2. Thus, higher success was achieved by creating a separate mechanism for caries. Vinayahalingam et al. [38] achieved high success (accuracy of 0.87) with the MobileNet V2 deep learning model, which they used to classify carious lesions in third molar tooth images cropped from 253 panoramic X-rays. Zhu et al. [40] segmented shallow, moderate and deep caries in panoramic X-rays with Caries Net deep learning architecture. They achieved a mean 93.64% Dice coefficient and 93.61% accuracy at three different caries levels. Haghanifar et al. [41] detected dental caries on (470) panoramic X-ray images close to those in our study and achieved 90.52% caries detection accuracy. In our study, 70.79% F1-score success was found in the detection of occlusal caries and 67.65% F1-score in the detection of proximal caries with the ResNet50-DCDNet learning architecture. Lian et al. [37] compared the caries depths classified by expert dentists on 1160 panoramic X-rays with the efficiency of the artificial intelligence program. As a result of the study, they found the performance of the artificial intelligence program and expert dentists to be similar.

Studies show that even experienced dentists are not consistent in diagnosing proximal caries [42]. When early lesions are missed, the chance to perform minimally invasive procedures may be lost. Li et al. [43] marked 953 pits and fissures in 712 intraoral photographs and 1002 approximal caries. While they found over 88% sensitivity in pit and fissure caries and approximal caries detected by the deep learning-based prototype artificial intelligence system, this compares with an over 67% F1-score in the same type of caries in our study. One of the reasons for the 18.64% F1-score success rate in the detection of cervical caries in our study may be that the proposed DCDNet architecture could not learn this type of caries adequately due to the insufficiency of the dataset in this type, which we call type III. Another reason may be that panoramic radiographs are 2D images and this type of caries is usually located in the vestibule and lingual of the teeth, so it overlaps with the denser healthy tooth tissue and, at the same time, it can be challenging to detect because of overlapping with the radiolucent reflection of the pulp chamber on the radiograph.

The limitations of this study are the insufficient dataset, especially for cervical caries. In addition, the panoramic radiographs used in the study could not be supported by clinical examination of the patients. As a result, dental caries whose cavitation has just begun to form in the mouth may have been overlooked in the panoramic radiographs and some dental caries detected by artificial intelligence may have been evaluated as false positive by experts when they were present in the mouth, and vice versa. At the same time, the superposition of the teeth, which is frequently seen in panoramic radiographs, may have caused some dental caries not to be detected.

It is indisputable that the use of artificial intelligence as an auxiliary diagnostic tool in dentistry will be of great benefit in reducing the workload of the dentist and in making an accurate diagnosis. There is a need for further studies on the use of artificial intelligence in different areas of dentistry, its possible negative effects and the development of artificial intelligence programs and deep learning methods that will enable higher levels of success to be achieved.

## 5. Conclusions

Within the limitations of this study, while the deep learning-based artificial intelligence system successfully detected occlusal and proximal caries, it showed low performance in detecting cervical caries. More powerful datasets and new network architectures could enable the detection of cavities in different locations with higher success rates. Thus, deep learning-based artificial intelligence systems may become one of the favorite elements in dental clinics by increasing the success rate of dentists in diagnosis and treatment and providing dentists with ease of work.

## Figures and Tables

**Table 1:** Backbone networks and skip connections used in the proposed DCDNet network.

Backbone	Skip Connection 1	Skip Connection 2	Skip Connection 3	Skip Connection 4
MobilenetV2	Block 13 expands relu	block 6 expand relu	block 3 expand relu	block 1 expand relu
Inception-V3	Mixed7	Mixed2	Activation 5	Activation 3
EfficientNet	block6a expand activation	block4a expand activation	block3a expand activation	block2a expand activation
ResNet50	stage4 unit1 relu1	stage3 unit1 relu1	stage2 unit1 relu1	relu0
VGG16	block5 conv3	block4 conv3	block3 conv3	block2 conv2

**Table 2:** Detailed layer structure and connections (layer inputs) of the DCDNet model (Sc: skip connection, f: filters, c: kernel, s: stride).

Layer Name	Section	Layer Input	Applied Layer Process	Details
L0	Encoder	Image	Pre-training Network	Details in Table 1
L1	MCF-level 0	Sc0	Pointwise Convolution	f: 256, c: 3 × 3, s = 1 × 1
L2		L1	Conv2DTranspose	f: 256, c: 4 × 4, s = 2 × 2
L3	MCF-level 1	Sc1	Pointwise Convolution	f: 256, c: 3 × 3, s = 1 × 1
L4		L2, L3	Concat	-
L5		L4	Conv2DTranspose	f: 256, c: 4 × 4, s = 2 × 2
L6		L4	Convolution, Batch Normalization, ReLU	f: 128, c: 3 × 3, s = 1 × 1
L7		L6	UpSampling2D	s = 8 × 8
L8	MCF-level 2	Sc2	Pointwise Convolution	f: 256, c: 3 × 3, s = 1 × 1
L9		L5, L8	Concat	-
L10		L9	Conv2DTranspose	f: 256, c: 4 × 4, s = 2 × 2
L11		L9	Convolution, Batch Normalization, ReLU	f: 128, c: 3 × 3, s = 1 × 1
L12		L11	UpSampling2D	s = 4 × 4
L13	MCF-level 3	Sc3	Pointwise Convolution	f: 256, c: 3 × 3, s = 1 × 1
L14		L10, L13	Concat	-
L15		L14	Conv2DTranspose	f: 256, c: 4 × 4, s = 2 × 2
L16		L14	Convolution, Batch Normalization, ReLU	f: 128, c: 3 × 3, s = 1 × 1
L17		L16	UpSampling2D	s = 2 × 2
L18	MCF-level 4	Sc4	Pointwise Convolution	f: 256, c: 3 × 3, s = 1 × 1
L19		L15, L18	Concat	-
L20		L19	Conv2DTranspose	f: 256, c: 4 × 4, s = 2 × 2
L21		L19	Convolution, Batch Normalization, ReLU	f: 128, c: 3 × 3, s = 1 × 1
L22	MPO	L6, L11, L16, L19	Concat	-
L23		L22	Convolution, Batch Normalization, ReLU	f: 32, c: 3 × 3, s = 1 × 1
L24		L22	Convolution, Batch Normalization, ReLU	f: 32, c: 3 × 3, s = 1 × 1
L25		L22	Convolution, Batch Normalization, ReLU	f: 32, c: 3 × 3, s = 1 × 1
L26	Output 1	L23	Convolution, Sigmoid	f: 1, c: 1 × 1, s = 1 × 1
L27	Output 2	L24	Convolution, Sigmoid	f: 1, c: 1 × 1, s = 1 × 1
L28	Output 3	L25	Convolution, Sigmoid	f: 1, c: 1 × 1, s = 1 × 1

**Table 3:** F1-score results obtained with different backbone networks of DCDNet architecture (%).

DCDNet Models	Type I (Occlusal Caries)	Type II (Proximal Caries)	Type III (Cervical Caries)	Weighted Averages of F1-Score
Mobilenet V2	73.45	66.15	11.21	61.86
Inception-V3	69.25	64.86	14.29	60.20
EfficientNet	71.93	68.01	12.90	62.67
ResNet50	70.79	67.65	18.64	62.79
VGG16	64.88	64.32	7.55	57.76

**Table 4:** Detailed results of the proposed models for Type I, Type II and Type III.

DCDNet Models	Metrics	Type I (Occlusal Caries)	Type II (Proximal Caries)	Type III (Cervical Caries)
MobilenetV2	Precision	76.02	71.48	20.00
	Recall	71.04	61.56	7.79
	F1-Score	73.45	66.15	11.21
Inception-V3	Precision	73.89	72.86	24.14
	Recall	65.17	58.45	10.14
	F1-Score	69.25	64.86	14.29
EfficientNet	Precision	75.93	74.58	37.50
	Recall	68.33	62.50	7.79
	F1-Score	71.93	68.01	12.90
ResNet50	Precision	72.00	70.55	29.73
	Recall	69.61	64.97	13.58
	F1-Score	70.79	67.65	18.64
VGG16	Precision	70.78	71.53	14.81
	Recall	59.89	58.43	5.06
	F1-Score	64.88	64.32	7.55

**Table 5:** F1-Score Performance comparison of DCDNet and other models.

Models	Type I (Occlusal Caries)	Type II (Proximal Caries)	Type III (Cervical Caries)	Weighted Averages of F1-Score
ResNet50-DCDNet	70.79	67.65	18.64	62.79
Unet	12.97	16.08	01.88	13.44
FPNet	14.68	11.02	02.24	11.10
Mobile-UNet	18.36	16.53	00.20	15.46
Eff-Unet	17.69	16.91	00.40	15.69

**Table 6:** Comparison of deep learning models in terms of time consumption (ms: milliseconds, s: seconds).

Models	FPS (ms)	FPS (s)
Mobile-UNet	18.9	0.0189
Unet	23.0	0.0230
Eff-Unet	33.0	0.0330
FPNet	37.5	0.0375
Mobilenet-DCDNet	80.0	0.0800
VGG16-DCDNet	85.7	0.0857
InceptionV3-DCDNet	97.3	0.0973
EfficientNet-DCDNet	97.9	0.0979
ResNet50-DCDNet	98.3	0.0983

## Data Availability

Data is available from the corresponding author upon reasonable request.
